# Influence of Technological Housing Conditions on the Concentration of Airborne Dust in Dairy Farms in the Summer: A Case Study

**DOI:** 10.3390/ani13142322

**Published:** 2023-07-16

**Authors:** Pavel Kic

**Affiliations:** Department of Technological Equipment of Buildings, Faculty of Engineering, Czech University of Life Sciences Prague, 16521 Prague, Czech Republic; kic@tf.czu.cz

**Keywords:** cattle, dairy cows, calf, Dust-Track, PM fractions, constructions, straw, ventilation

## Abstract

**Simple Summary:**

Due to modern housing systems and new barn concepts, an indoor environment on dairy farms has become very important. Airborne dust is a significant pollutant in the indoor environment of animal barns. An increased concentration of small airborne dust particles can cause health problems for farm animals and workers. This research shows that cattle barns with straw bedding cause more indoor airborne dust than barns without straw. Increased ventilation airflow has a positive effect on reducing the concentration of airborne dust particles, which was especially evident in the comparison of older, massive brick cowsheds compared to modern, light, uninsulated barns. The installation and operation of axial fans with a horizontal axis of rotation significantly help natural ventilation in terms of airborne dust concentration reduction. The results of the research showed that a large proportion of airborne dust particles in the summer are made up of the smallest dust particles.

**Abstract:**

This research shows the size composition of airborne dust fractions in selected dairy barns down to the smallest particles, including factors that influence this composition. Measurements with a Dust-Track 8530 laser photometer took place in the summer at external temperatures of 29.5 to 36 °C. In barns with straw bedding, the average total dust concentration TDC was 66.98 ± 28.38 μg·m^−3^ (PM_10_ 60.11 ± 19.93 μg·m^−3^, PM_4_ 49.48 ± 13.76 μg·m^−3^, PM_2.5_ 44.78 ± 10.18 μg·m^−3^, and PM_1_ 38.43 ± 9.29 μg·m^−3^). In barns without straw bedding, the average TDC was 55.91 ± 36.6 μg·m^−3^, PM_10_ 33.71 ± 13.86 μg·m^−3^, PM_4_ 30.69 ± 15.29 μg·m^−3^, PM_2.5_ 27.02 ± 13.38 μg·m^−3^, and PM_1_ 22.93 ± 10.48 μg·m^−3^. The largest TDC of 108.09 ± 32.93 μg·m^−3^ (PM_10_ 69.80 ± 18.70 μg·m^−3^, PM_4_ 68.20 ± 18.41 μg·m^−3^, PM_2.5_ 53.27 ± 14.73 μg·m^−3^, and PM_1_ 38.46 ± 5.55 μg·m^−3^) was measured in an old cowshed with stanchion housing for 113 cows, straw bedding, and ventilation through windows. In a modern cowshed for loose housing of 440 lactating cows without straw bedding, with natural ventilation and 24 axial fans, TDC was 53.62 ± 49.52 μg·m^−3^, PM_10_ 20.91 ± 5.24 μg·m^−3^, PM_4_ 17.11 ± 3.23 μg·m^−3^, PM_2.5_ 13.71 ± 0.92 μg·m^−3^, and PM_1_ 12.69 ± 2.82 μg·m^−3^. In all investigated barns, a large proportion of airborne dust particles (54.38 ± 20.82% of TDC) consists of the smallest PM_1_ dust particles (from 12.69 ± 2.82 μg·m^−3^ to 48.48 ± 1.18 μg·m^−3^).

## 1. Introduction

Considerable attention has long been paid to stressful situations and conditions for farm animals [1,2]. Animals’ welfare can be threatened by many factors; exposure to adverse climatic conditions can also lead to stress conditions [3].

The indoor environment in animal houses is characterized by a significant biological load, which causes a large production of harmful substances. In terms of air cleanliness, in addition to gaseous pollutants, airborne dust is an important pollutant. The concentration of airborne dust is greatly influenced by the animal species, the housing technology, the size of the barn, and the number of housed animals [4,5,6,7,8,9,10]. The highest airborne dust concentration is found in barns for poultry and pigs; in sheds for cattle, it is usually lower [4,5,6,7,8,9]. Therefore, research on the cleanliness of the indoor environment and emissions from livestock buildings pays less attention to airborne dust in buildings for cattle [11].

The number of people who develop asthma and allergies grows each year [12]. Research [13] shows the severity of cattle farm allergies in young farmers. Research on farms [14] showed allergy symptoms related to cattle breeding (upper respiratory tract, asthma, and skin). Due to the nature of farm work, the greatest risk to farmers’ health is biological hazards in the form of numerous microorganisms and their metabolites, plant particles, and animal particles contained in organic dust [15,16].

Published studies on dust exposure among livestock farmers show that the workforce in these industries is overexposed and at risk of developing respiratory diseases. Thus, there is an urgent need to focus on exposure in agriculture to protect farmers and others working in these and related industries from developing respiratory diseases and allergies [17].

Airborne dust has a harmful effect on the health of workers [4,6,7,8,9,18,19], but it can also have a negative effect on housed animals [4,5,6,7,8,9,20,21,22,23,24,25]. In terms of their effect on the organism, dust particles in animals’ houses are classified as airborne dust without a fibrogenic effect but with an irritating effect. The irritating effect can affect not only the respiratory tract but also the mucous membranes of the eyes or skin. The irritating effect is caused by their composition, as they are dust particles of animal origin (feathers, wool, fur, and other animal dust) and plant origin (hay, straw, grain, feed mixtures, etc.) [4,5,6,7,8,9].

Considerable attention is paid to issues of microbiology in terms of nutrition, production quality, and animal health [20,21,22]. Microorganisms (bacteria, fungi, fungal spores, etc.) are also abundant in the barn dust, whose effect on the health of the organism can be harmful [4,5,6,7,8,9,11,26,27,28,29,30].

The size of the airborne dust particles is important. From the entire size spectrum of solid particles, particles over 100 μm are removed very quickly by gravity. Smaller particles, and especially respirable particles, remain in the air longer [31,32,33,34]. Since the health risks and penetration of particles into the respiratory tract depend on their aerodynamic properties, particles are classified according to them.

Large airborne dust particles, which tend to settle more quickly, are therefore not as significant a threat to humans as fine particles [31,32,33,34,35,36,37,38]. Small particles can stay in the air longer and can easily enter the respiratory tract. Therefore, a classification is introduced that divides airborne dust particles based on their size and the depth of penetration into the respiratory system.

Airborne dust particles are divided into fractions [32,33,34,35]:

− Inhalable fraction: occurs in the air and is inhaled through the nose or mouth. If the size of dust particles exceeds 10 μm, then such particles are attached mainly in the upper respiratory tract;
− Thoracic fraction: of the total inhaled airborne dust, the fraction that penetrates beyond the larynx after inhalation;
− Respirable fraction: entering areas of the respiratory system that do not have cilia-type epithelium. Such particles do not exceed 4 μm; they penetrate into the alveoli and are therefore very dangerous.

To clarify measurement and assessment methods, in addition to the total airborne dust mass concentration (TDC), dust particles are divided according to size into PM_10_, PM_4_, PM_2.5,_ and PM_1_ fractions.

The respirable fraction is taken in the event of airborne dust with a predominantly fibrogenic effect. The sources of airborne dust in barns are primarily housed animals, feed, and bedding, and therefore airborne dust in barns is primarily organic [4,5,6,7,8,9], which does not pose a risk of fibrogenic effects. Determining other fractions may be justified for research and special tasks.

Occupational exposure limits (OELs) are regulatory values (concentration limits) that indicate levels of exposure that are considered to be safe (health-based) for a hazardous contaminant in the air of a workplace.

According to [35], this type of airborne dust has only irritating effects (poultry feathers, particles of feed, straw, and sawdust). Occupational Exposure Limits important for animal houses are for animal dust: feathers (4 mg·m^−3^), wool, fur, and other animal dust (6 mg·m^−3^), for straw, and cereals (6 mg·m^−3^).

Measured airborne dust inside this type of building is not aggressive; therefore, as a criterion for comparative evaluation of the measured values, it could be interesting to use the limit level of outdoor airborne dust. According to the Air Protection Act No. 201/2012 [37], the PM_10_ limit value in 24 h is 50 µg·m^−3^, the 1-year limit value is 40 µg·m^−3^, and the 1-year limit value for PM_2.5_ is 25 µg·m^−3^. According to [38], the limit level for PM_10_ is 50 µg·m^−3^ for a 24 h exposure period and 25 µg·m^−3^ for an annual exposure period; the limit level for PM_2.5_ is 25 µg·m^−3^ for a 24 h exposure period and 8 µg·m^−3^ for an annual exposure period.

According to [6,38,39], measurement of the PM_1_ fraction is important and should also have been considered in monitoring programs and possibly in future regulations because the magnitude of this fraction might not be negligible. Thus, differential particle sampling is encouraged, even going down to the ultra-fine fraction. In summary, research should address particle composition and sources, particle size, PM levels, and the factors influencing these.

The time of day or night and the technological operations and activities that take place in the animals’ houses also influence the concentration of airborne dust in the buildings [4,40,41,42,43]. Dust concentration will increase significantly, e.g., during bedding or feeding [4,8,9,40,41,42,43,44]. The season of the year and high air humidity also influence airborne dust concentration [4,41,43]. Higher airborne dust concentrations were therefore found inside the buildings for farm animals in the winter, and researchers from northern Europe pay a lot of attention to measuring airborne dust [4,10,27,28,41,43].

Light airborne dust particles also spread to the surroundings of farms. Research on airborne dust on cattle farms is therefore conducted not only inside the barns but also in the paddock during the summer [45].

The experiments with dairy cows were conducted in an air-conditioned cowshed. The effect of the regulated ionic microclimate on the emission of PM_10_ dust particles was positive, and airborne dust concentration was slightly reduced [46].

The aim of this research is to demonstrate that the size composition of airborne dust fractions in dairy cattle barns is important to examine in more detail, down to the smallest particles, including the factors that influence this composition.

## 2. Materials and Methods

### 2.1. Description of Buildings Used for Research

This research was carried out on several farms located in the Czech Republic. The Czech Republic lies in a mild climate zone that is characterized by the alternation of four seasons. Due to its location in the central part of the European mainland, the influence of the ocean and the westerly direction of airflow prevail here [47]. In the territory of the Czech Republic, the long-term monthly average air temperature has a simple annual pattern with a minimum mainly in January and a maximum mainly in July and August. The average air temperature in the Czech Republic from 1961 to 1970 in July was 16.7 °C and in August 15.8 °C, and from 2012 to 2021 in July it was 19.9 °C and in August 18.3 °C [48]. Relative air humidity ranges from 60–80% on an annual average. It is significantly influenced by daily temperatures and the amount of precipitation. The lowest is usually between April and August.

Airborne dust concentration was measured during the summer when no technological operations such as milking, feeding, cleaning, or bedding with straw were taking place in the barns.

For this research, all barns investigated are in the same climatic region, typical for agricultural conditions. The buildings for dairy cattle housing were selected in such a way that the causes of possible differences in the airborne dust concentrations inside the investigated buildings could be identified and shown. Barns thus represent the most common types of agricultural buildings for housing dairy cows and other categories of cattle in the Czech Republic; however, the building structure, building materials, and technological equipment for housing differ. The numbers of housed animals are also different, which, however, also corresponds to their use in the operation of dairy cattle farms.

The research included the measurement and evaluation of indoor airborne dust in buildings: dairy production cowsheds for lactating cows LC (number of housed animals inside the building): LC 100, LC 113, LC 116, LC 210, LC 440, barn for dry cows DC 23, cowshed for maternity cows MC 27, calf hutch CaH 1, barn for calves CaB 36, and for comparison, also in one barn with beef cows and calves BC 38.

Summary information about the researched barns and selected construction and operational parameters is processed in Table 1.

Dairy production cowsheds for lactating cows (LC 100) are used for housing 100 Jersey cows in cubicles with straw bedding. The roof with a central ridge slot is covered by plastic plates, partly completed by translucent fiberglass; gable walls are partly made from wood and partly from PVC mesh; side walls are made from PVC mesh, which can be covered by the vertically movable PVC tarpaulin.

Old cowshed LC 113, with 113 Holstein dairy cows, has stanchion housing with straw bedding. Ventilation is through slightly open windows and doors. In addition to dairy cows, calves are also housed in the barn in the first period after giving birth for approximately 2 weeks, near the lactating dairy cow—their mother.

Old cowshed for lactating cows LC 116 with a capacity of 116 dairy cows is constructed from massive stone and brick walls. It is used for housing 80 Jersey cows in individual cubicles with straw bedding. The natural ventilation of the building is through partially open windows and doors.

Modern cowshed LC 210 with loose housing technology for 210 Holstein lactating cows has comfort cubicles covered by separated dried manure solids as bedding. Longitudinal walls are made of woven fabric mesh and variable side curtains, which, together with a ventilation roof ridge slot, enable natural ventilation. Eight fans improve the ventilation of the building in the summer.

Dairy production cowshed LC 440 for Holstein lactating cows is a new modern cowshed for loose housing of lactating cows. It is a semi-closed building with comfort cubicles covered by separated dried manure solids as bedding. The walls are made of woven fabric mesh with variable side curtains and a roof ridge slot for natural ventilation. If the air temperature is over 24 °C, the installed 24 axial fans switch automatically to improve ventilation.

The cowshed DC 23 for dry Holstein cows is a massive brick structure. It has an outdoor feeding passage and a rest area with loose housing on deep litter. The central slot of the roof ridge and the open windows allow ventilation. In summer, ventilation is improved by four axial fans.

Cowshed for maternity Holstein cows (MC 27) are used for housing 27 dairy cows before and at the time of calving. The maternity cowshed has free housing with straw bedding in nine maternity pens. Natural ventilation is provided by the ridge roof and mesh side walls.

Individual outdoor hutches, CaH 1, are used for Holstein calves in the first breeding period (2 months). It is made of white polyethylene. CaH has a length of 150 cm, a width of 112 cm, and a height of 135 cm. The floor is covered daily with straw bedding.

Barn for calves CaB 36 is a modern, spacious wooden building for housing 36 Jersey calves during the milk feeding period. Calves are in individual pens with straw bedding. The barn has sufficient natural ventilation thanks to the large open areas of the side walls, which are open in the summer and covered only with mesh.

Barn for Angus beef cattle BC 38 is a reconstructed brick cowshed (originally for dairy cows) used for beef cows with calves and beef cattle fattening (during the measurement of 38 animals) in group pens with littered lying areas. The natural ventilation of the barn is provided by partly open windows in the walls and a ridge roof slot.

### 2.2. Data Acquisition and Processing

Temperatures and relative humidity of the air were measured by instruments and sensors from the Ahlborn company (Ahlborn Mess- und Regelungstechnik GmbH, Eichenfeldstraße 1, 83607 Holzkirchen, Germany) outside and inside the buildings with registration at intervals of 1 min.

The Almemo 2690 with temperature and humidity sensors of the FHA 646 for outside measurement, together with the FY A600 sensor for CO_2_ measurement, was kept in a special weather station box.

The Almemo 2590-9 with sensors FHA 646 and FY A600 were used inside the barns near the animals. Temperature and humidity sensors of the FHA 646 type have a range of use from −20 to 80 °C and from 5 to 98% relative humidity. The measuring range of the capacitive sensor of relative humidity ranges from 0 to 100%, with an accuracy of ±2% in the range <90% relative humidity. The concentration of CO_2_ was measured by the Ahlborn sensors FY A600, with an operative range of 0 to 0.5% and an accuracy of ±0.01%.

The total dust mass concentration was measured by the special exact laser-photometer instrument Dust-Track™ II Aerosol Monitor 8530 produced by TSI in the USA, 500 Cardigan Road, Shoreview, MN 55126, with an operating range of 0.001 to 150 mg·m^−3^ and a resolution of ±0.1% of the reading of 0.001 mg·m^−3^, whichever is greater. Zero calibration was used before every use.

Size-selective impactors can be attached to the inlet of the Dust-Track™ II Aerosol Monitor 8530. Size-selective impactors are used to pre-condition the size range of the particles entering the instrument. PM_1_, PM_2.5_, PM_4,_ and PM_10_ impactors were used to measure segregated mass fractions of airborne dust.

For the purpose of this measurement, 90 airborne dust concentration data points were collected for the total airborne dust concentration as well as for each PM size fraction of dust in each measured building. The measurement frequency was 2 s. Measurements were taken sequentially for each barn during warm summer days. The Dust-Track™ II device was always placed in a representative location of the investigated building at a height of 0.6 m above the floor.

The acquired datasets were processed using MS Excel, and some of the results (assessing whether differences between evaluated datasets are significant or not) were verified by the statistical software TIBCO SW Data Science Workbench Statistica Version 6 (*ANOVA* and *Tukey’s HSD (Honestly Significant Difference) test*). Data processed in the form of charts was processed in MS Excel.

## 3. Results

The average temperatures and relative humidity of the outdoor air at the time of airborne dust measurement are summarized in Table 2. The average temperatures of the outdoor air ranged from 29.5 ± 0.2 °C to 36.0 ± 0.1 °C, and the average relative humidity ranged from 16.5 ± 0.2% to 40.3 ± 0.1%.

Table 2 also shows the corresponding temperatures, relative humidity, and CO_2_ concentration of the indoor air in the buildings at the time of airborne dust measurement. Average indoor air temperatures ranged from 27.2 ± 0.2 °C to 43.3 ± 0.1 °C, and average relative humidity ranged from 15.4 ± 0.1% to 50.3 ± 2.9%.

The CO_2i_ concentration inside the barns at the time of measurement in half of the buildings corresponded to the outdoor CO_2e_ concentration, which was 400 ppm. In four buildings, the concentration of CO_2i_ was slightly increased, ranging from 410 ± 0 ppm to 583 ± 171 ppm; in one barn, it was 939 ± 132 ppm.

The airborne dust measurement results are summarized in Table 3. In addition to the total dust mass concentration (TDC), the concentrations of individual fractions PM_10_, PM_4_, PM_2.5,_ and PM_1_ are also shown in Table 3 for individual buildings.

The measured results of total dust mass concentration show significant differences between some buildings. Total dust mass concentration was the highest in cowshed LC 113 (108.09 ± 32.93 μg·m^−3^), an old cowshed with stanchion housing and straw bedding.

The use of plenty of bedded straw and limited ventilation was also manifested in the older brick barns DC 23 (86.81 ± 33.59 μg·m^−3^) and BC 38 (76.33 ± 17.72 μg·m^−3^). The difference in total dust mass concentration between them was not statistically significant.

Between cowshed LC 100 (60.22 ± 7.00 μg·m^−3^), calf hutch CaH 1 (58.63 ± 6.27 μg·m^−3^), cowshed LC 210 (58.2 ± 14.76 μg·m^−3^), cowshed LC 116 (57.29 ± 12.58 μg·m^−3^), cowshed LC 440 (53.62 ± 49.52 μg·m^−3^), and barn for calves CaB 36 (53.02 ± 4.82 μg·m^−3^), there was no statistically significant difference in total dust mass concentration. However, there are barns with and without straw bedding and significant differences in housing technology, as well as in terms of the concentration of housed animals inside buildings.

Total dust mass concentration was the lowest in maternity cowshed MC 27 (37.59 ± 13.74 μg·m^−3^) for housing cows before and after calving. A large amount of straw bedding is used in the pens, but the pens for the cows are spacious, and the natural ventilation through the mesh walls, supplemented by fans, has a very good effect on the cleanliness of the inside air.

There are also significant differences in the assessment of the PM_10_ dust fraction between some barns. The highest PM_10_ was in the barn for dry cows DC 23 (86.50 ± 23.70 μg·m^−3^); there was no significant difference between the barn for beef cattle BC 38 (73.99 ± 12.62 μg·m^−3^) and the cowshed LC 113 (69.80 ± 18.70 μg·m^−3^). A little lower PM_10_ was in cowshed LC 100 (58.04 ± 5.86 μg·m^−3^) and calf hutch CaH 1 (54.81 ± 5.18 μg·m^−3^), but there was no significant difference between calf hutch CaH 1 (54.81 ± 5.18 μg·m^−3^), cowshed LC 116 (52.67 ± 4.16 μg·m^−3^), and barn for calves CaB 36 (52.13 ± 3.39 μg·m^−3^).

Lower values of PM_10_ were measured in the cowshed without straw bedding LC 210 (46.51 ± 5.38 μg·m^−3^), the maternity cowshed MC 27 (30.86 ± 4.09 μg·m^−3^), and significantly lower compared to other barns in the large-capacity cowshed without straw bedding LC 440 (20.91 ± 5.24 μg·m^−3^).

In the assessment of PM_4_, the highest airborne dust level was in the cowshed, with the highest total dust mass concentration (with stanchion housing and straw bedding) of LC 113 (68.20 ± 18.41 μg·m^−3^). The lower fraction of PM_4_ was in the group of barns: cowshed LC 100 (55.01 ± 2.39 μg·m^−3^), barn for beef cattle BC 38 (52.42 ± 3.20 μg·m^−3^), barn for calves CaB 36 (49.11 ± 1.30 μg·m^−3^), and cowshed LC 116 (48.84 ± 1.61 μg·m^−3^). There was no statistically significant difference in PM_4_ between the calf hutch CaH 1 (48.09 ± 1.88 μg·m^−3^), the barn for dry cows DC 23 (47.90 ± 14.68 μg·m^−3^), and the cowshed LC 210 (44.27 ± 9.41 μg·m^−3^). Significantly lower PM_4_ was in maternity cowshed MC 27 (26.27 ± 1.84 μg·m^−3^) and the smallest PM_4_ was in large-capacity cowshed LC 440 without straw bedding (17.11 ± 3.23 μg·m^−3^).

In the evaluation of the dust fraction PM_2.5_, the worst situation was in cowshed LC 100 (53.53 ± 1.98 μg·m^−3^) and cowshed LC 113 (53.27 ± 14.73 μg·m^−3^). Lower PM_2.5_ was in the cowshed LC 116 (48.79 ± 2.39 μg·m^−3^) and barn for calves CaB 36 (47.56 ± 0.82 μg·m^−3^); there was no significant difference between the calf hutch CaH 1 (45.47 ± 1.13 μg·m^−3^) and barn for beef cattle BC 38 (44.56 ± 0.82 μg·m^−3^). There was no significant difference between the cowshed for lactating cows LC 210 (40.32 ± 1.81 μg·m^−3^) and the cowshed for dry cows DC 23 (40.09 ± 2.99 μg·m^−3^). A very low value of PM_2.5_ was in the maternity cowshed MC 27 (25.01 ± 1.18 μg·m^−3^), the significantly lowest of all buildings in the modern cowshed LC 440 (13.71 ± 0.92 μg·m^−3^).

The concentration of the smallest airborne dust particles, PM_1_, was statistically very significantly different between almost all barns. The PM1 dust fraction was the highest in cowshed LC 100 (48.48 ± 1.18 μg·m^−3^); it was slightly lower in barns: barn for calves CaB 36 (45.78 ± 1.17 μg·m^−3^) and cowshed LC 116 (45.72 ± 0.84 μg·m^−3^); there was no significant difference between them. The other barns differed statistically significantly from each other in terms of PM_1_, as is clear from Table 3. The lowest PM_1_ fraction was the in large-capacity cowshed without straw bedding, LC 440 (12.69 ± 2.82 μg·m^−3^).

In order to compare barns with and without straw bedding and to find out whether the use of straw has a significant effect on total dust mass concentration and how it affects individual fractions PM_10_, PM_4_, PM_2.5,_ and PM_1_, sets of measured results of barns with straw bedding (LC 113, DC 23, BC 38, LC 100, CaH 1, LC 116, CaB 36, and MC 27) and without straw bedding (LC 210 and LC 440) were compared.

This comparison of the two sets shown in Table 4 shows what the differences are and where the conditions are better from this point of view. In individual columns, the different superscript letters mean that there is a significant difference between the values in the column.

Table 5 shows the results of a statistical comparison of the average airborne dust levels (total dust mass concentration, PM_10_, PM_4_, PM_2.5,_ and PM_1_) in solid brick constructions (masonry) with straw bedding, ventilated only by slightly open windows and doors (LC 113, DC 23, BC 38, and LC 116), with lightweight sheds also with straw bedding, the walls of which are only covered with nets (LC 100, CaB 36, and MC 27).

Table 6 shows the results of a statistical comparison of average dust levels in barns with natural ventilation supplemented for better ventilation with axial fans with a horizontal axis of rotation (DC 23, LC 210, LC 440, MC 27) and barns ventilated only by natural ventilation without fans (LC 113, BC 38, LC 100, CaH 1, LC 116, and CaB 36).

Table 7 shows the results of a statistical comparison of the average dust levels in cowsheds with separately housed cows (DC 23, LC 100, LC 210, LC 116, and LC 440), with barns only for calves (CaH 1 and CaB 36), and with barns in which cows are housed together with calves (LC 113, BC 38, and MC 27).

**Figure 1:**
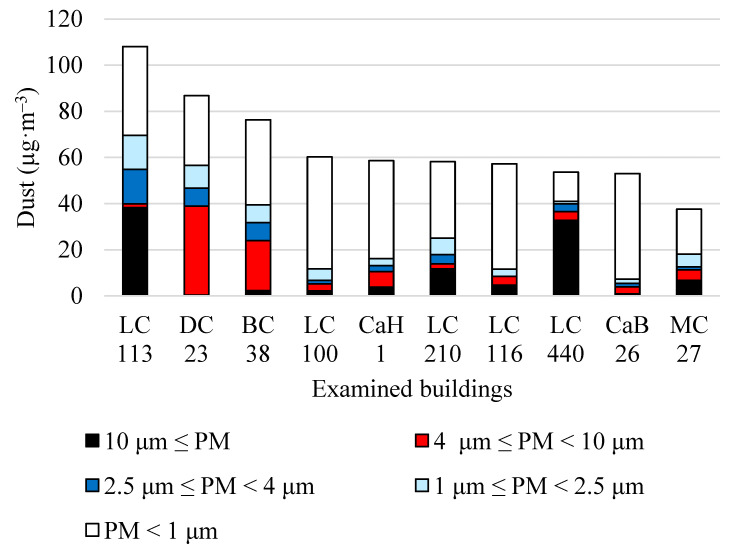
The average mass concentrations (μg·m^−3^) of airborne dust particles in the examined buildings are in the range above 10 μm, from 4 μm to 10 μm, from 2.5 μm to 4 μm, from 1 μm to 2.5 μm, and smaller than 1 μm.

Figure 1 is a chart showing summary information on the total dust mass concentration of airborne dust. Each column shows 5 subcomponents of individual size groups of airborne dust particles in the range above 10 μm, from 4 μm to 10 μm, from 2.5 μm to 4 μm, from 1 μm to 2.5 μm, and smaller than 1 μm. The order of the columns in the picture corresponds to the order according to the average size of the total dust mass concentration in barns. The measured values of airborne dust particle fractions are given in mass concentration (μg·m^−3^).

**Figure 2:**
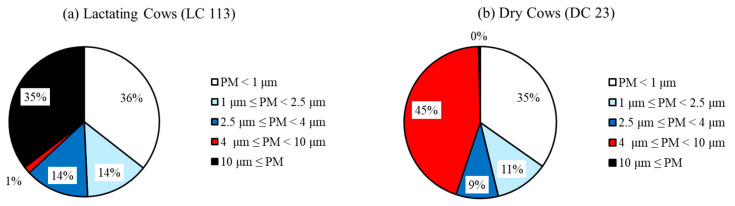

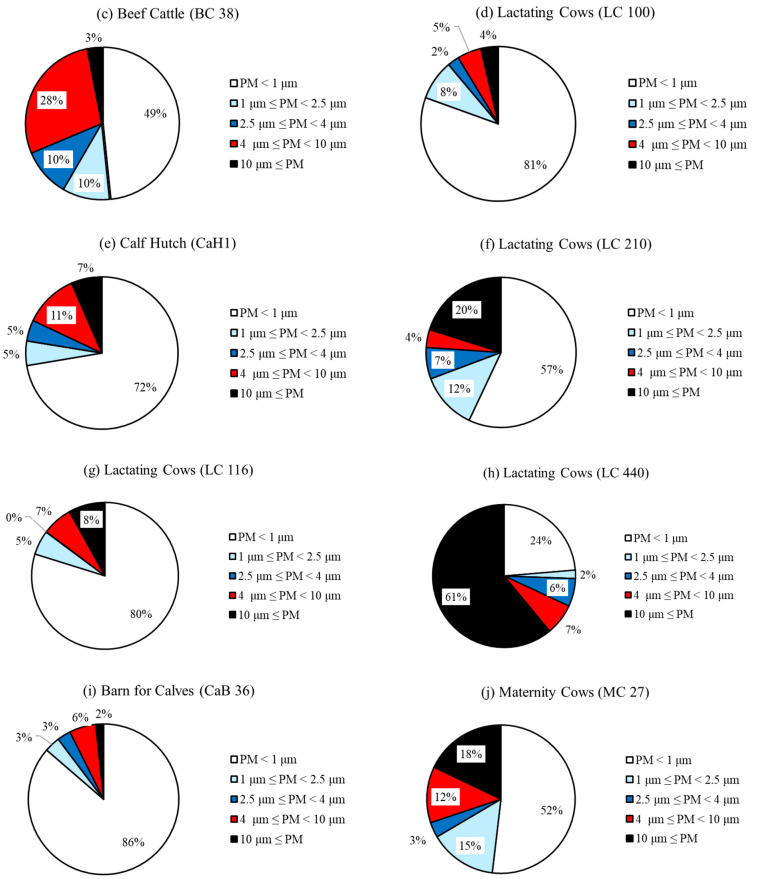
The average concentrations of airborne dust particles in all examined buildings: (**a**) LC 113; (**b**) DC 23; (**c**) BC 38; (**d**) LC 100; (**e**) CaH 1; (**f**) LC 210; (**g**) LC 116; (**h**) LC 440; (**i**) CaB 36; (**j**) MC 27. The share of the size distribution of dust particle fractions inside the buildings in the range above 10 μm, from 4 μm to 10 μm, from 2.5 μm to 4 μm, from 1 μm to 2.5 μm, and smaller than 1 μm is in.

The share of individual fractions of airborne dust particles in the air in individual barns is rather variable. The share of the size distribution of airborne dust particle fractions PM ˂ 1 μm, 1 μm ≤ PM ˂ 2.5 μm, 2.5 μm ≤ PM ˂ 4 μm, 4 μm ≤ PM ˂ 10 μm, 10 μm ≤ PM as a percentage of total dust mass concentration in the examined barns is shown in Table 8 and Figure 2.

## 4. Discussion

Previous studies have reported higher airborne dust concentrations in poultry and pig barns compared to cattle barns [4,5,6,7,8,9,10]. As a rule, however, more detailed attention was not paid to the differences in the method of housing, the construction, or the ventilation system of the barn. Under the conditions of this study, it is possible to look in more detail at the differences in the construction of the barns as well as the housing and ventilation solutions.

The results of extensive measurements [42] in animal houses for poultry, pigs, cattle, and mink showed the lowest concentrations of airborne dust in cattle houses. According to the data in [8], in the barns for cattle, the concentration of airborne dust PM_10_ was 100 μg·m^−3^, and PM_2.5_ only 10 μg·m^−3^. The results in Table 3 show that in this research, PM_10_ was lower than 100 μg·m^−3^ in all barns, and PM_2.5_ was always higher than 10 μg·m^−3^.

The measurement method using the laser-photometer Dust-Track™ II Aerosol Monitor 8530 has the advantage of using impactors for detecting PM dust fractions, including the smallest particles, and is very suitable for comparative measurements between different objects [42].

All measurements in this study were made in the same climatic area during the hot summer season (see Table 2). However, certain differences in outdoor temperature and relative humidity are caused by air temperature fluctuations during the summer. It is practically impossible to achieve the same outdoor parameters for all measurements.

The internal temperatures and air humidity are also slightly different, which is also caused by the different massiveness of the structures, different thermal-technical properties of the investigated buildings, different methods of ventilation, different housing technologies, different excrement removal, etc.

The results in Table 3 show significant differences between individual cattle barns in terms of the selected criteria for evaluating dust concentration. The division and evaluation of the researched barns into different groups (categories) according to several aspects (straw bedding vs. without straw bedding; massive structures vs. light uninsulated sheds; natural ventilation only vs. natural ventilation supplemented with fans) will help to reveal the reserves of the currently existing state and find possibilities for possible improvement of the current situation.

A comparison of the dust measurement results in Table 4 shows that in all measured airborne dust parameters, i.e., total dust mass concentration, PM_10_, PM_4_, PM_2.5_, and PM_1_, the average concentration of dust particles in barns with straw bedding was statistically significantly higher than in barns without straw bedding.

The results in Table 5 show that the total dust mass concentration and the concentration of PM_10_, PM_4_, and PM_2.5_ dust particles were significantly higher in the massive constructions with window and door ventilation than in the light barns, where natural ventilation is easier thanks to the walls partially covered only by nets. There was an insignificant difference in the concentration of the smallest dust particles in PM_1_.

The results in Table 6 show that the total dust mass concentrations of PM_10_, PM_4_, PM_2.5,_ and PM_1_ are significantly lower in barns equipped with fans for better ventilation in the summer than in barns that only have natural ventilation and are not equipped with fans. The effect of fans is statistically significant for reducing the total concentration of dust and all size fractions of dust.

The results in Table 7 show that in barns with cows and calves housed together, total dust mass concentration was significantly higher (74.00 ± 36.88 μg·m^−3^) than in cowsheds with cows housed separately (62.79 ± 30.74 μg·m^−3^) and by separately housed calves (55.83 ± 6.26 μg·m^−3^).

No statistically significant difference was found in the assessment of airborne dust concentration according to PM_10_ between the assessed groups of barns, but PM_10_ exceeded the limit of 50 μg·m^−3^ in all cases.

When evaluating PM_4_, the value was significantly lower in cowsheds with separately housed dairy cows (PM_4_ = 42.63 ± 15.47 μg·m^−3^) than in barns with calves (PM_4_ = 48.60 ± 1.70 μg·m^−3^) or in barns with cows housed together with calves (PM_4_ = 48.96 ± 20.41 μg·m^−3^), between which there was no significant difference.

Also, in the assessment of PM_2.5_, the value was lower in cowsheds with separately housed dairy cows (PM_2.5_ = 39.29 ± 13.94 μg·m^−3^), but it was not statistically significantly different from barns with cows and calves housed together (PM_2.5_ = 40.94 ± 14.63 μg·m^−3^), which was, however, significantly less than in barns with separate calves (PM_2.5_ = 46.51 ± 1.44 μg·m^−3^).

When evaluating the concentration of the smallest dust particles of PM_1_, there was a statistically significant difference between all evaluated groups of barns. The worst was PM_1_ in barns with calves (PM_1_ = 44.10 ± 1.98 μg·m^−3^), the lowest was in cowsheds with cows (PM_1_ = 34.05 ± 12.88 μg·m^−3^), and the lowest was PM_1_ in common barns with dairy cows and calves (PM_1_ = 31.63 ± 9.21 μg·m^−3^).

In all measured buildings, a large part (54.38 ± 20.82%) of the smallest PM_1_ dust particles were present in the air. This can be attributed to the fact that these smallest and lightest dust particles move freely in the flowing air, and their settling time is very long, so even a low airspeed keeps them in the air. Part of these small dust particles is removed by ventilation, and part is, on the other hand, swirled inside the space and lifted from the surfaces back into the air in the barn.

The percentage of the smallest PM_1_ particles is significantly lower in barns equipped with fans than in barns ventilated only by natural ventilation, as can be seen from the statistical evaluation in Table 6.

The smallest share of PM_1_ particles (24%) was in the large-capacity cowshed without straw bedding (LC 440). It is also the smallest in terms of absolute values (PM_1_ = 12.69 ± 2.82 μg·m^−3^). Conversely, the largest share (61%) of the largest dust particles above 10 μm was found in this cowshed, LC 440. This is mainly due to the higher airflow speed inside this cowshed. In addition to the natural flow through the mesh walls, 24 axial fans with a horizontal axis of rotation, which help to better ventilate the barn in the summer, are the source of higher flow speeds inside the barn. The PM_1_ particles are removed by air streams, and the largest particles above 10 μm recirculate inside the barn.

According to the authors [19], organic dust consists mostly of particles with a diameter of less than 1 μm, which is unacceptable for long-term exposure and can be the cause of asthma, asthmatic syndromes, chronic bronchitis, and hypersensitivity pneumonitis (Farmer’s lung). According to the results in Table 3 and Table 8 and Figure 1 and Figure 2, it is clear that in this aspect our research and conclusions agree.

In the overall evaluation according to Table 8, the largest particles, 10 μm ≤ PM, are the second fraction of dust particles in percentage order (15.94 ± 18.29%).

The third size fraction of dust particles (4 μm ≤ PM < 10 μm) accounted for 13.70 ± 12.78% of the total dust concentrations in the barns.

The fourth group of dust particles consists of the size fraction 1 μm ≤ PM < 2.5 μm, which had a share of 9.07 ± 4.25%.

The smallest part of the total concentration of dust in the investigated stables was made up of particles 2.5 μm ≤ PM ˂ 4 μm, which were 6.91 ± 3.94%.

The authors [9] report that the average PM_10_ in dairy cattle farms (cubicle housing without straw bedding) was 40 μg·m^−3^, ranging from 14 to 95 μg·m^−3^, and the average PM_2.5_ was 13.8 μg·m^−3^, ranging from 3.9 up to 24.9 μg·m^−3^. The results of PM_10_ measured in cowsheds without litter (33.71 ± 13.86 μg·m^−3^), see Table 3, correspond approximately to these average values; the results of PM_10_ found in barns with straw bedding (60.11 ± 19.93 μg·m^−3^) correspond to the upper half of the range according to [9]. PM_2.5_ values found in cowsheds without straw bedding (27.02 ± 13.38 μg·m^−3^) exceed the data according to [9]. They are even more significantly exceeded by the results from barns with straw bedding (44.78 ± 10.18 μg·m^−3^); see Table 4.

The authors of the research [19] on small dairy farms state that the measured values of PM_10_ during the distribution of hay and feed flour did not exceed the limit of 5 mg·m^−3^ for organic dust, but in some individual cases, they exceeded the value of 10 mg·m^−3^. The stated values are thus significantly higher than the results found by this research, presented in Table 3 and others.

Research results in Finnish cubicle cow houses [41] indicate mean concentrations of total airborne dust of 0.2–1.9 μg·m^−3^, which is significantly more than the results of the measurements of this research in the summer period, presented in Table 3. Measurements [41] were made during winter and early autumn, when the indoor temperatures and relative humidity varied from 7 to 17 °C (mean 12.7 °C) and 55 to 95% (mean 87%), respectively. The outdoor temperature ranged from +2 to −20 °C. The external and internal conditions differ considerably from those of this research (see Table 2). Due to the cold measurement conditions, the CO_2_ concentration results were also higher in the Finnish stables than in the investigated barns of this case study in the Czech Republic in the summer.

Concentrations of airborne particulate matter, ammonia, and carbon dioxide in large-scale uninsulated loose-housing cowsheds in Estonia were measured from September 2008 to August 2009 [43]. The mean recorded concentrations of PM_total_ were 205 ± 270 μg·m^−3^, PM_10_ 65 ± 121 μg·m^−3^, PM_2.5_ 18 ± 46 μg·m^−3^, and PM_1.0_ 10 ± 11 μg·m^−3^. The overall mean inside air CO_2_ concentration was 553 ± 315 ppm. The mean air temperature was 9.6 ± 6.6 °C, and the relative humidity was 83.2 ± 16.8%.

The results of measuring total mass dust concentration TDC from 37.59 ± 13.74 μg·m^−3^ to 108.09 ± 32.93 μg·m^−3^ achieved in this research (Table 3) are higher in [43] (TDC corresponds to PM_total_). Also, the PM_10_ values in this research, from 30.86 ± 4.09 μg·m^−3^ to 69.80 ± 18.70 μg·m^−3^ are lower than in [43]. On the contrary, the average values found during this research (Table 3) for PM_2.5_ from 25.01 ± 1.18 μg·m^−3^ to 53.27 ± 14.73 μg·m^−3^ and PM_1_ from 19.50 ± 0.82 μg·m^−3^ to 38.46 ± 5.55 μg·m^−3^ are higher than in [43].

The low mean air temperatures and high relative air humidity measured in [43] correspond to the colder climatic regions of Estonia; however, this comparison is interesting for the mutual comparison of airborne dust concentration and airborne dust individual fractions. It can be assumed that the high concentrations of PM_2.5_ and PM_1_ fractions achieved in this research (Table 3) are caused by high temperatures and low relative air humidity when measured in the summer period in the Czech Republic.

If it is an assessment of the cleanliness of the air in the barn from the point of view of occupational health [35], the occupational exposure limits important for animal houses, which are for feather (4 mg·m^−3^), wool, fur, and other animal dust (6 mg·m^−3^), straw, and cereals (6 mg·m^−3^), were not exceeded in any measurement, even in the case of total dust mass concentration results.

Considering the current trend in livestock breeding to strive for the most favorable animal welfare, which will be close to the natural conditions of animals kept in the wild, it is interesting to compare the internal environmental conditions in the barns in terms of air pollution by airborne dust with the limits recommended for the stay of people in the outdoor environment. However, these data are also interesting from the point of view of the stay of people in the relevant premises as part of their work activities and duties.

When comparing the measured values with the limit value of recommended air quality standards for PM_10_ = 50 μg·m^−3^ for the outdoor environment for a 24 h exposure period [31,32,33,34,35], the lower value was only in cowshed without straw bedding LC 210 (46.51 ± 5.38 μg·m^−3^) and maternity cowshed MC 27 (30.86 ± 4.09 μg·m^−3^), and significantly lower compared to other barns was in large-capacity cowshed without straw bedding LC 440 (20.91 ± 5.24 μg·m^−3^). This cowshed was the only one of the investigated barns that would also meet the requirement of PM_10_ = 25 μg·m^−3^ for an annual exposure period. The requirement for an annual exposure period [37] of PM_10_ = 40 μg·m^−3^ would also be met for cowshed MC 27.

The recommended air quality standards are slightly different. According to [37] PM_2.5_ = 20 μg·m^−3^ for an annual exposure period, according to [38] PM_2.5_ = 8 μg·m^−3^ for an annual exposure period, and PM_2.5_ = 25 μg·m^−3^ for a 24-h exposure period. The PM_2.5_ value = 8 μg·m^−3^ for an annual exposure period was exceeded in all barns. The value of PM_2.5_ = 20 μg·m^−3^ was not exceeded only in cowshed LC 440 (PM_2.5_ = 13.71 ± 0.92 μg·m^−3^), and the limit value of PM_2.5_ = 25 μg·m^−3^ was reached by the maternity cowshed MC 27 (25.01 ± 1.18) μg·m^−3^.

## 5. Conclusions

From the results presented in the tables, it follows that dairy cattle barns equipped with laying stalls with straw bedding cause higher indoor air dust pollution than barns without straw bedding.

A comparison of the measurement results presented in the tables showed that increased ventilation has a positive effect on reducing the concentration of airborne dust particles.

A comparison of the measurement results presented in the tables shows that in modern, uninsulated cowsheds with a large proportion of open walls made of woven fabric mesh and variable side curtains, the concentration of airborne dust was lower than in modernized massive brick barns with a smaller proportion of open parts of the building, which would allow ventilation.

From the results presented in tables and figures, it follows that in the summer season, a large proportion of airborne dust particles in most dairy cattle barns are the smallest PM_1_ particles, regardless of the type of construction and technology of housing with straw bedding or without straw. For this reason, attention should also be paid to these smallest airborne dust particles in further research.

## Figures and Tables

**Table 1:** Main characteristics of the investigated barns.

Measured Object	Construction	Housing	Area m^2^·Animal^−1^	Straw kg·Animal^−1^·d^−1^	Ventilation
LC 100	Steel; mesh	Cubicles	2.8	2–2.5	Natural
LC 113	Brick walls	Stanchion-tied	2.5	4	Windows; doors
LC 116	Stone and brick	Cubicles	2.8	2–2.5	Windows; doors
LC 210	Steel; mesh	Cubicles	3	-	Natural + fans
LC 440	Steel; mesh	Cubicles	3	-	Natural + fans
DC 23	Brick walls	Group pens	10	3.5	Natural + fans
MC 27	Steel; mesh	Group pens	15	5	Natural
CaH 1	Polyethylene	Hutch	1.7	1.5	Natural
CaB 36	Timber	Individual pens	3.4	2	Natural
BC 38	Brick walls	Group pens	9	5	Natural

**Table 2:** Basic parameters of the outside air and inside the barns at the time of airborne dust measurement.

Measured Object	t_e_ °C ± SD	RH_e_% ± SD	t_i_ °C ± SD	RH_i_% ± SD	CO_2i_ ppm
LC 100	35.9 ± 0.1	16.7 ± 0.3	32.7 ± 0.1	27.4 ± 0.3	400 ± 0
LC 113	29.5 ± 0.2	40.0 ± 0.1	27.2 ± 0.2	50.3 ± 2.9	939 ± 132
LC 116	36.0 ± 0.1	16.5 ± 0.2	30.7 ± 0.1	30.2 ± 0.4	410 ± 0
LC 210	29.8 ± 0.1	40.3 ± 0.1	28.1 ± 0.8	44.2 ± 1.8	573 ± 16
LC 440	31.4 ± 0.5	33.1 ± 1.2	29.8 ± 0.1	43.4 ± 1.7	583 ± 171
DC 23	32.9 ± 0.3	29.3 ± 1.0	32.7 ± 0.5	34.0 ± 0.8	503 ± 74
MC 27	32.3 ± 0.2	30.1 ± 0.4	33.6 ± 0.2	30.0 ± 0.7	400 ± 0
CaH 1	33.3 ± 0.2	19.5 ± 0.2	43.3 ± 0.1	15.4 ± 0.1	400 ± 0
CaB 36	33.9 ± 0.1	19.0 ± 0.4	33.7 ± 0.0	22.6 ± 0.1	400 ± 0
BC 38	32.8 ± 0.7	32.7 ± 1.3	29.8 ± 0.3	44.5 ± 0.1	400 ± 0

SD—standard deviation.

**Table 3:** Results of measurement and statistical comparison of total dust mass concentration (μg·m^−3^) and concentrations of fractions (μg·m^−3^) PM_10_, PM_4_, PM_2.5,_ and PM_1_ in all investigated buildings. Different superscript letters (^a, b, c, d, e, f, g, h, i^) are a sign of a highly significant difference (*ANOVA*; *Tukey HSD test*; *p ≤ 0.01*) between the measured values in the individual columns.

Measured Object	TDC ± SD	PM_10_ ± SD	PM_4_ ± SD	PM_2.5_ ± SD	PM_1_ ± SD
LC 100	60.22 ± 7.00 ^c^	58.04 ± 5.86 ^c^	55.01 ± 2.39 ^c^	53.53 ± 1.98 ^a^	48.48 ± 1.18 ^d^
LC 113	108.09 ± 32.93 ^a^	69.80 ± 18.70 ^a^	68.20 ± 18.41 ^a^	53.27 ± 14.73 ^a^	38.46 ± 5.55 ^a^
LC 116	57.29 ± 12.58 ^c^	52.67 ± 4.16 ^d^	48.84 ± 1.61 ^bc^	48.79 ± 2.39 ^e^	45.72 ± 0.84 ^g^
LC 210	58.20 ± 14.76 ^c^	46.51 ± 5.38 ^e^	44.27 ± 9.41 ^bd^	40.32 ± 1.81 ^b^	33.18 ± 1.35 ^f^
LC 440	53.62 ± 49.52 ^c^	20.91 ± 5.24 ^f^	17.11 ± 3.23 ^e^	13.71 ± 0.92 ^f^	12.69 ± 2.82 ^h^
DC 23	86.81 ± 33.59 ^b^	86.50 ± 23.70 ^b^	47.90 ± 14.68 ^b^	40.09 ± 2.99 ^b^	30.20 ± 1.21 ^b^
MC 27	37.59 ± 13.74 ^d^	30.86 ± 4.09 ^g^	26.27 ± 1.84 ^f^	25.01 ± 1.18 ^g^	19.50 ± 0.82 ^i^
CaH 1	58.63 ± 6.27 ^c^	54.81 ± 5.18 ^cd^	48.09 ± 1.88 ^bd^	45.47 ± 1.13 ^cd^	42.42 ± 0.92 ^e^
CaB 36	53.02 ± 4.82 ^c^	52.13 ± 3.39 ^de^	49.11 ± 1.30 ^bc^	47.56 ± 0.82 ^de^	45.78 ± 1.17 ^g^
BC 38	76.33 ± 17.72 ^b^	73.99 ± 12.62 ^a^	52.42 ± 3.20 ^c^	44.56 ± 0.82 ^cd^	36.92 ± 1.06 ^c^

SD—standard deviation.

**Table 4:** Results of measurement and statistical comparison of total dust mass concentration (μg·m^−3^) and concentrations of fractions (μg·m^−3^) PM_10_, PM_4_, PM_2.5,_ and PM_1_ in barns with straw bedding (LC 113, DC 23, BC 38, LC 100, CaH 1, LC 116, CaB 36, and MC 27) and without straw bedding (LC 210 and LC 440). Different superscript letters (^a, b^) are a sign of a significant difference (*ANOVA*; *Tukey HSD test; p ≤ 0.01*) between the measured values in the individual columns.

Bedding	TDC ± SD	PM_10_ ± SD	PM_4_ ± SD	PM_2.5_ ± SD	PM_1_ ± SD
Straw	66.98 ± 28.38 ^a^	60.11 ± 19.93 ^a^	49.48 ± 13.76 ^a^	44.78 ± 10.18 ^a^	38.43 ± 9.29 ^a^
Without Straw	55.91 ± 36.6 ^b^	33.71 ± 13.86 ^b^	30.69 ± 15.29 ^b^	27.02 ± 13.38 ^b^	22.93 ± 10.48 ^b^

SD—standard deviation.

**Table 5:** Results of measurement and statistical comparison of total dust mass concentration (μg·m^−3^) and concentrations of fractions (μg·m^−3^) PM_10_, PM_4_, PM_2.5,_ and PM_1_ in solid brick constructions (masonry) with straw bedding (LC 113, DC 23, BC 38, and LC 116) and lightweight sheds with straw bedding, the walls of which are only covered with nets (LC 100, CaB 36, and MC 27). Different superscript letters (^a, b^) are a sign of a significant difference (*ANOVA*; *Tukey HSD test; p ≤ 0.01*) between the measured values in the individual columns.

Construction	TDC ± SD	PM_10_ ± SD	PM_4_ ± SD	PM_2.5_ ± SD	PM_1_ ± SD
Masonry	82.13 ± 31.75 ^a^	70,72 ± 20.47 ^a^	54.34 ± 14.45 ^a^	46.68 ± 9.11 ^a^	37.83 ± 6.24 ^a^
Lightweight	49.55 ± 12.57 ^b^	47.74 ± 13.38 ^b^	43.46 ± 12.54 ^b^	42.03 ± 12.36 ^b^	37.92 ± 13.11 ^a^

SD—standard deviation.

**Table 6:** Results of measurement and statistical comparison of total dust mass concentration (μg·m^−3^) and concentrations of fractions (μg·m^−3^) PM_10_, PM_4_, PM_2.5,_ and PM_1_ in barns with natural ventilation supplemented with axial fans (DC 23, LC 210, LC 440, and MC 27) and barns ventilated only by natural ventilation without fans (LC 113, BC 38, LC 100, CaH 1, LC 116, and CaB 36). Different superscript letters (^a, b^) are a sign of a significant difference (*ANOVA*; *Tukey HSD test; p ≤ 0.01*) between the measured values in the individual columns.

Ventilation	TDC ± SD	PM_10_ ± SD	PM_4_ ± SD	PM_2.5_ ± SD	PM_1_ ± SD
Natural and fans	59.06 ± 36.23 ^a^	46.19 ± 27.99 ^a^	33.89 ± 15.51 ^a^	29.78 ± 11.32 ^a^	23.89 ± 8.41 ^a^
Natural	68.57 ± 25.34 ^b^	60.59 ± 13.19 ^b^	53.61 ± 10.43 ^b^	48.86 ± 7.14 ^b^	42.96 ± 4.82 ^b^

SD—standard deviation.

**Table 7:** Results of measurement and statistical comparison of total dust mass concentration (μg·m^−3^) and concentrations of fractions (μg·m^−3^) PM_10_, PM_4_, PM_2.5,_ and PM_1_ in cowsheds with separately housed cows (DC 23, LC 100, LC 210, LC 116, and LC 440), with barns only for calves (CaH 1 and CaB 36), and with barns in which cows are together with calves (LC 113, BC 38, and MC 27). Different superscript letters (^a, b, c^) are a sign of a highly significant difference (*ANOVA*; *Tukey HSD test*; *p ≤ 0.01*) between the measured values in the individual columns.

Housed Category of Cattle	TDC ± SD	PM_10_ ± SD	PM_4_ ± SD	PM_2.5_ ± SD	PM_1_ ± SD
Cows	62.79 ± 30.74 ^a^	53.36 ± 24.21 ^a^	42.63 ± 15.47 ^a^	39.29 ± 13.94 ^a^	34.05 ± 12.88 ^a^
Calves	55.83 ± 6.26 ^a^	53.47 ± 4.58 ^a^	48.60 ± 1.70 ^b^	46.51 ± 1.44 ^b^	44.10 ± 1.98 ^b^
Cows and calves	74.00 ± 36.88 ^b^	58.19 ± 23.50 ^a^	48.96 ± 20.41 ^b^	40.94 ± 14.63 ^a^	31.63 ± 9.21 ^c^

SD—standard deviation.

**Table 8:** The share of the size distribution of dust particle fractions PM ˂ 1 μm, 1 μm ≤ PM ˂ 2.5 μm, 2.5 μm ≤ PM ˂ 4 μm, 4 μm ≤ PM ˂ 10 μm, 10 μm ≤ PM in the examined barns as a percentage of total dust mass concentration.

Airborne Dust Fractions	PM < 1 μm	1 μm ≤ PM < 2.5 μm	2.5 μm ≤ PM < 4 μm	4 μm ≤ PM < 10 μm	10 μm ≤ PM
Mean % ± SD	54.38 ± 20.82	9.07 ± 4.25	6.91 ± 3.94	13.70 ± 12.78	15.94 ± 18.29

SD—standard deviation.

## Data Availability

The farmers gave consent for the raw data to be used by the researcher only, but any queries involving the processing of these data can be directed to the corresponding author.
